# Supplementary material for: Dysmagnesemia in critically ill diarrheal patients in Bangladesh
Source: PLoS One. 2025 May 19;20(5):e0323467. doi: 10.1371/journal.pone.0323467 (PMC12088048; doi:10.1371/journal.pone.0323467)
Supplement: Supplementary Table 1 — (DOCX) [file pone.0323467.s001.docx]

**Supplementary Table 1: Outcome between Normomagnesemia vs Dysmagnesemia (either hypo or hypermagnesemia)**

| **Variables** | **Normal (n=132)** | **Hypomagnesemia (n=233)** | | | **Hypermagnesemia (n=23)** | | |
| --- | --- | --- | --- | --- | --- | --- | --- |
|  |  | **n (%)** | **OR**  **(95% CI)** | **p-value** | **n (%)** | **OR**  **(95% CI)** | **p-value** |
| **Required Mechanical Ventilation** | 10 (7.6) | 11 (4.7) | 0.6 (0.25, 1.46) | 0.265 | 3 (13.0) | 1.83 (0.46, 7.23) | 0.265 |
| **ICU stay (day),**  **Mean (SD)** | 1.3 ± 0.7 | 1.4 ± 0.8 | 1.2 (0.88, 1.64) | 0.241 | 1.2 ± 0.4 | 0.68 (0.28, 1.67) | 0.241 |
| **Outcome (discharge)** | 72 (54.6) | 117 (50.2) | Reference | | 12 (52.2) | Reference | |
| **LAMA or referred** | 50 (37.9) | 104 (44.6) | 1.28 (0.82, 2.00) | 0.279 | 8 (34.8) | 0.96 (0.37, 2.52) | 0.279 |
| **Death** | 10 (7.6) | 12 (5.2) | 0.74 (0.30, 1.80) | 0.504 | 3 (13.0) | 1.80 (0.43, 7.5) | 0.504 |

OR: odds ratio; CI: confidence interval; LAMA: left against medical advice
